# Impaired autonomic function in adolescents born preterm

**DOI:** 10.14814/phy2.13620

**Published:** 2018-03-29

**Authors:** Kristin Haraldsdottir, Andrew M. Watson, Kara N. Goss, Arij G. Beshish, David F. Pegelow, Mari Palta, Laura H. Tetri, Gregory P. Barton, Melissa D. Brix, Ryan M. Centanni, Marlowe W. Eldridge

**Affiliations:** ^1^ Department of Pediatrics University of Wisconsin Madison Wisconsin; ^2^ Department of Kinesiology University of Wisconsin Madison Wisconsin; ^3^ Department of Orthopedics & Rehabilitation University of Wisconsin Madison Wisconsin; ^4^ Department of Medicine University of Wisconsin Madison Wisconsin; ^5^ Department of Biostatistics and Medical Informatics University of Wisconsin Madison Wisconsin

**Keywords:** Autonomic function, exercise physiology, heart rate recovery, heart rate variability

## Abstract

Preterm birth temporarily disrupts autonomic nervous system (ANS) development, and the long‐term impacts of disrupted fetal development are unclear in children. Abnormal cardiac ANS function is associated with worse health outcomes, and has been identified as a risk factor for cardiovascular disease. We used heart rate variability (HRV) in the time domain (standard deviation of RR intervals, SDRR; and root means squared of successive differences, RMSSD) and frequency domain (high frequency, HF; and low frequency, LF) at rest, as well as heart rate recovery (HRR) following maximal exercise, to assess autonomic function in adolescent children born preterm. Adolescents born preterm (less than 36 weeks gestation at birth) in 2003 and 2004 and healthy age‐matched full‐term controls participated. Wilcoxon Rank Sum tests were used to compare variables between control and preterm groups. Twenty‐one adolescents born preterm and 20 term‐born controls enrolled in the study. Preterm‐born subjects had lower time‐domain HRV, including SDRR (69.1 ± 33.8 vs. 110.1 ± 33.0 msec, respectively, *P* = 0.008) and RMSSD (58.8 ± 38.2 vs. 101.5 ± 36.2 msec, respectively, *P* = 0.012), with higher LF variability in preterm subjects. HRR after maximal exercise was slower in preterm‐born subjects at 1 min (30 ± 12 vs. 39 ± 9 bpm, respectively, *P* = 0.013) and 2 min (52 ± 10 vs. 60 ± 10 bpm, respectively, *P* = 0.016). This study is the first report of autonomic dysfunction in adolescents born premature. Given prior association of impaired HRV with adult cardiovascular disease, additional investigations into the mechanisms of autonomic dysfunction in this population are warranted.

## Introduction

With great advances in neonatal care over the past three decades, survival is improving for the lowest gestational age and birthweight infants (Ruegger et al. 2012). As neonatal outcomes improve, there has been increased interest in understanding the long‐term effects of premature birth on multiple organ systems. In fact, the NIH now recommends that premature birth be considered a long‐term medical condition, though the long‐term implications are only beginning to be understood. (Bhutta et al. 2002). Children and young adults born preterm are smaller, with lower height and weight than term‐born controls (Rogers et al. 2005), are frequently less physically active (Rogers et al. 2005; Lowe et al. 2016), have higher blood pressure (Bertagnolli et al. 2016), abnormalities in the renin‐angiotensin system (South et al. 2017), and tend to have reduced exercise capacity (Kilbride et al. 2003; Rogers et al. 2005; Smith et al. 2008; Takken et al. 2010).

The mechanisms behind these deficits in children born preterm are unclear, but may be in part explained by the interrupted neural development that occurs during fetal development. The autonomic nervous system (ANS) develops significantly in the third trimester of fetal development (Schneider et al. 2009), a time period where premature birth occurs. The ANS unconsciously controls body functions such as breathing, blood pressure regulation, temperature regulation, and cardiac function. ANS activity has been shown to be lower in infants born preterm, with a strikingly greater impact on the parasympathetic arm, where lower gestational age at birth is associated with lower parasympathetic activity in infancy up to 6 months of age (Patural et al. 2004; Landrot et al. 2007). Although data regarding autonomic function in individuals who survive beyond infancy is limited, one study demonstrated that children 6–7 years old have no difference in heart rate variability and ANS function from term‐born controls (Landrot et al. 2007), suggesting that there may be “catch‐up growth” in the ANS in early childhood. Interestingly, a study in young adults born premature demonstrates evidence of autonomic dysfunction, specifically reduced parasympathetic regulatory capacity (Mathewson et al. 2015), which may either suggest that there is a re‐emergence of autonomic dysfunction in young adulthood, or that there is continuous autonomic dysfunction from preterm birth that was not captured by the limited research in children.

In this study, we employed two noninvasive measures of cardiovascular autonomic function, heart rate variability (HRV) and heart rate recovery (HRR). HRV is a noninvasive tool to measure the variability in the R‐R interval in an electrocardiography (ECG) reading, where greater variability in the R‐R intervals reflects greater autonomic activity (Berntson et al. 1997). HRR following maximal exercise is largely influenced by the reactivation of vagal tone on the atria of the heart, and the reduction in sympathetic tone to the heart (Savin et al. 1982). Faster HRR is correlated with greater autonomic function, and slower HRR has been correlated with cardiovascular disease risk (Cole et al. 1999). Here, we sought to investigate autonomic function in adolescent children aged 12–14 born very premature who are otherwise healthy and free of current respiratory or cardiovascular disease. The aim of the study was to determine whether autonomic function is altered in adolescents born preterm using a resting HRV measurement, and a HRR period following maximal exercise testing.

## Methods

### Ethical approval

The protocol was approved by the Institutional Review Boards at the University of Wisconsin Madison. Each subject was informed of the purpose and risks associated with the study and written consent was obtained from all subjects and a legal guardian in accordance with the standards set by the Declaration of Helsinki.

### Participants

Preterm participants were recruited from the Newborn Lung Project (Palta and Sadek‐Badawi 2008; Palta et al. 2008), a cohort established at the University of Wisconsin (Madison, WI) that enrolled individuals born preterm (≤36 weeks gestation) with very low birth weight (<1500 g) in 2003 and 2004 in Wisconsin. The Newborn Lung Project also included normal birth weight (NBW) term‐born children whose addresses were obtained from 2003 to 2004 Wisconsin birth records (Palta and Sadek‐Badawi 2008), from which 14 controls were recruited. The remaining control subjects were recruited from the local community using flyers. Inclusion criteria for all participants was ability to complete a maximal exercise test, nonsmoking, free of mental, physical, visual or neurological disabilities, and no diagnosed current cardiovascular or respiratory disease. Subjects’ height was measured using a mechanical measuring rod to the nearest 0.5 cm (Seca; Hamburg, Germany), and weight was measured using a digital scale to the nearest 0.1 kg (Taylor; Oak Brook, IL) and recorded at the beginning of the study visit.

### Baseline physical activity questionnaire

Subjects completed a physical activity questionnaire, the PAQ‐C, an externally validated and widely used physical activity quantification tailored for children, to determine physical activity level. Questions were based on a 7‐day recall of low intensity to high intensity activities during school, immediately following school, and in the evening (Crocker et al. 1997).

### Resting heart rate variability

Heart rate variability measurements were obtained in a subset of the study population, 13 control and 12 preterm subjects. In these subjects, a continuous 15‐min 3‐lead ECG recording (PowerLab 16/30, LabChart version 8.0, ADInstruments, Colorado Springs, CO) was obtained prior to any other study activities. Subjects lay supine on a bed in a dark and quiet room, instructed to lie still for the entire 15‐min data collection period. The time domain variables considered in this study were the mean RR interval and its standard deviation (SDRR), representing overall HRV and its root mean square successive difference (RMSSD), representing the vagal tone, and pRR50, the percentage of differences higher than 50 msec in RR intervals (DeGiorgio et al. 2010). The frequency‐domain variables were determined offline using the heart rate variability software package by PowerLab and was analyzed via LabChart software (ADInstruments, Inc., Colorado Springs, CO). HRV in the frequency domain was determined using the low frequency (LF) and high frequency (HF) cutoffs of 0.04–0.15 Hz and 0.15–0.40 Hz, respectively, where the LF power is mediated by both sympathetic and parasympathetic activity, and HF power is mediated by parasympathetic activity (Rakow et al. 2013). HF and LF values were submitted to natural log transformations to normalize their distributions (ln ms^2^) before analysis (Mathewson et al. 2015). Resting HR was obtained from this period and was determined as the lowest 30‐second average during the resting period.

### Graded exercise testing

Participants performed an incremental maximal exercise test on an upright cycle ergometer, with continuous wattage controlled (Velotron; RacerMate; Seattle, WA) while breathing room air. Participants cycled at 60–70 revolutions per minute (rpm) starting at 50 W for 2 min, and wattage increased by 25 W every 2 min until subjects were no longer able to maintain 55 rpm for more than 5‐sec, despite strong verbal encouragement. Heart rate was continuously monitored using forehead pulse oximetry (OxiMax N‐595; Nellcor, Mansfield, MA), expired gases were collected in a breath‐by‐breath manner (Gemini; CWE, Ardmore, PA) and ventilatory and metabolic parameters were continuously recorded and analyzed in PowerLab (ADInstruments; Colorado Springs, CO). Heart rate, ventilatory, and metabolic parameters were recorded and analyzed in PowerLab (ADInstruments, Colorado Springs, CO). Maximal oxygen consumption (VO_2max_) was determined from a rolling 30‐sec average. In order for a test to be considered a valid VO_2max_, the primary criteria of a plateau in VO_2_ defined as a change in <2 mL/kg/min in O_2_ consumption over the last 60 sec of the test had to be met, in addition to one of the following secondary criteria: (1) a maximal heart rate (HR_max_) of more than 90% age predicted HR_max_ (220‐age), or (2) a respiratory exchange ratio (carbon dioxide production/oxygen consumption) of ≥1.1.(Midgley et al. 2007) Oxygen pulse was calculated as the VO_2max_ in ml/min divided by HR_max_. VO_2max_ was reported both absolutely (L/min) and relative to body weight (mL/kg/min).

### Heart rate recovery

After reaching maximal volitional exhaustion, subjects were instructed to stop pedaling and sit completely still and quietly on the bicycle while HR was recorded for 2 min. HRR was calculated as the absolute drop in HR from HR_max_ for 2 min at 10‐second intervals (HRR_abs_). To adjust for wide ranges in HR_max_, HRR at 1 and 2 min was also calculated as the percentage of HR_max_ that had been recovered, by dividing HRR_abs_ by HR_max_ and multiplying by 100 (HRR_%max_).

### Statistical analysis

Wilcoxon Rank Sum tests were used to compare demographic, autonomic and metabolic variables between the control and preterm groups and Cohen's d was calculated to determine the effect size (Cohen 1988), where a higher number indicates a greater effect size. Heart rate recovery was compared between groups at each 10‐second time point during the 2 min of recovery using separate Wilcoxon Rank‐Sum tests. Multiple pairwise comparisons were adjusted using the method previously described by Holm (Holm 1979). Data analyses were conducted with GraphPad Prism software (Version 7, GraphPad Software Inc., La Jolla, CA). All tests were two‐tailed and *P *<* *0.05 was used to define statistical significance.

## Results

### Characteristics of the subjects at baseline

Twenty‐one preterm adolescents (age 13.0 ± 0.7 years, 27.9 ± 2.1 weeks gestation at birth) and twenty term‐born adolescents (age 13.3 ± 0.7 years, 39.9 ± 0.8 weeks gestation at birth) completed the study. Adolescents born preterm were shorter in stature, but no statistically significant differences were identified with respect to BMI z score or percentile (Table 1).

**Table 1:** Anthropometric and birth status data

	Control (*n* = 20)	Preterm (*n* = 21)	*P*‐value	Cohen's d
Female *n*, %	11, 55%	13, 62%		
Age (years)	13.3 ± 0.7	13.0 ± 0.7	0.094	0.567
Height (cm)	164.3 ± 8.0	158.1 ± 8.7	0.029	0.754
Weight (kg)	52.4 ± 10.5	46.6 ± 8.4	0.064	0.628
BMI (kg/m^2^)	19.4 ± 3.8	18.6 ± 2.6	0.415	0.273
BMI percentile (%)	47 ± 32	43 ± 29	0.678	0.098
BMI *z*‐score	−0.07 ± 1.15	−0.25 ± 0.95	0.58	0.123
BSA (m^2^)	1.56 ± 0.18	1.42 ± 0.17	0.013	0.86
Birthweight (grams)	3497 ± 366	1097 ± 274	<0.001	7.71
Gestational age (weeks)	39.7 ± 0.9	27.9 ± 2.1	<0.001	8.2
PAQ‐C	1.93 ± 0.39	1.89 ± 0.45	0.762	0.106

All data are expressed as mean ± SD. BMI, body mass index; BSA, body surface area; PAQ‐C, physical activity questionnaire children's.

### Physical activity

No statistically significant differences were identified between groups with respect to physical activity as reported on the PAQ‐C (Table 1).

### Exercise capacity

VO_2max_ was significantly lower in adolescents born preterm than controls when expressed as absolute liters of oxygen consumed per minute, but the difference was no longer statistically different when adjusted for body mass. Maximal oxygen pulse was significantly lower in preterms than controls (Table 2).

**Table 2:** Exercise capacity

	Control (*n* = 20)	Preterm (*n* = 21)	*P*‐value	Cohen's d
VO_2max_ (L/min)	2.47 ± 0.52	2.03 ± 0.47	0.011	0.854
VO_2max_ (mL/kg/min)	48.29 ± 11.01	43.32 ± 6.92	0.107	0.469
O_2_ pulse max (mL O_2_/beat)	12.77 ± 2.55	10.75 ± 2.67	0.023	0.796

All data are expressed as means ± SD. VO_2max_, maximal oxygen consumption; O_2_ pulse max, maximal oxygen consumed per heartbeat.

### Heart rate variability

**Figure 1:**
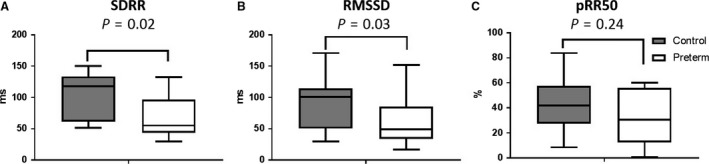
Resting heart rate variability. Standard deviation of R‐R intervals, SDRR. B. Root means squared of successive differences, RMSSD. Percentage of differences higher than 50 msec in RR intervals, pRR50. Box and whisker plots describe the mean of the data for each group, with bars showing the max and min values. Data are from 13 control and 12 preterm subjects.

Resting heart rates during the ECG period were similar between groups (Table 1). Heart rate variability in the time domain, represented by SDRR and RMSSD, was significantly lower in preterm subjects, with no difference in pRR50 between groups (Fig. 1). In the frequency domain, preterm subjects had higher LF heart rate variability, with no significant difference between groups in HF variability or the LF/HF ratio (Table 3).

**Table 3:** Autonomic measurements

	Control (*n* = 20)	Preterm (*n* = 21)	*P*‐value	Cohen's d
Resting HR pulse ox.	73.2 ± 13.2	75.0 ± 14.53	0.768	0.125
Resting HR ECG	74.1 ± 12.9	75.0 ± 14.5	0.878	0.066
LF (ms^2^)^a^	6.06 ± 0.86	6.91 ± 0.99	0.038	0.653
HF (ms^2^)^a^	6.10 ± 1.26	7.12 ± 1.21	0.056	0.826
LF/HF^a^	1.22 ± 0.95	1.29 ± 0.55	0.838	0.132
HR_max_	193.0 ± 9.5	192.9 ± 9.7	0.963	0.015
HRR_1 min_	39 ± 9	30 ± 12	0.013	0.860
HRR_2 min_	60 ± 10	52 ± 10	0.016	0.834
HRR%max_1 min_	20.4 ± 5.3	16.4 ± 6.5	0.05	0.667
HRR%max_2 min_	31.0 ± 5.7	27.1 ± 5.5	0.043	0.867

All data are expressed as mean ± SD. HR, heart rate; ECG, electrocardiogram; LF, low frequency heart rate variability; HF, high frequency heart rate variability; HR_max_, maximal heart rate; HRR_1 min_, heart rate recovery after 1 min of recovery; HRR_2 min_, heart rate recovery after 2 min of recovery; HRR%max_1 min_, heart rate recovery as a percentage of maximal heart rate after 1 min of recovery; HRR%max_2 min_, heart rate recovery as a percentage of maximal heart rate after 2 min of recovery.

a HRV frequency analysis was performed in 13 controls and 12 preterms.

### Heart rate recovery

**Figure 2:**
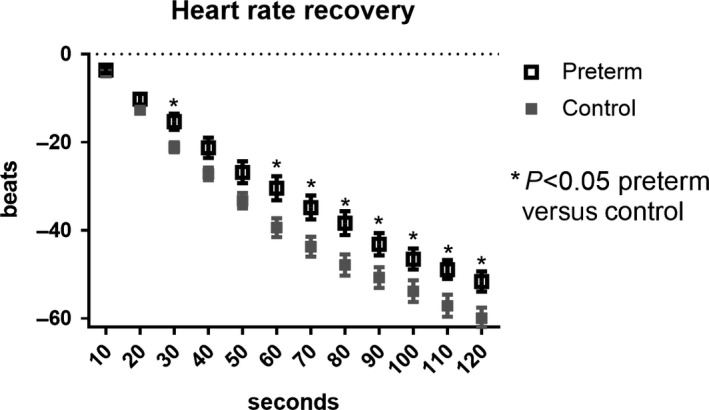
Heart rate recovery following maximal exercise. HRR in control (gray squares) and preterm (open squares) groups. Data are expressed as mean ± SEM. *P < 0.05 adjusted for pairwise comparison between control and preterm groups at each time point.

Heart rate recovery after maximal exercise was slower in preterm adolescents than controls throughout recovery (Table 3), with the difference achieving statistical significance at 30 sec through 2 min (Fig. 2). HRR as a percentage of maximal HR was significantly lower in preterm subjects at 1 and 2 min of recovery (Table 3).

## Discussion

In this study, we sought to determine whether cardiac autonomic function is altered in adolescent children born preterm. Our preterm population was shorter in stature, weighed less, but had similar BMI z‐scores and physical activity scores. Our study evaluated healthy children with a history of very premature birth, where none of the children had known respiratory or cardiovascular disease, and had no issues performing graded maximal exercise testing to exhaustion. Using noninvasive techniques, our resting and recovery autonomic function results suggest that otherwise healthy adolescent children born very premature have evidence of autonomic dysfunction using two separate measures. Preterm individuals had slower heart rate recovery following maximal exercise, lower heart rate variability at rest in the time domain, with elevated LF variability. We found that HRV is lower in preterm children. The significantly lower time domain HRV and blunted HRR following exercise suggest abnormal cardiac ANS activity in 12‐ to 14‐year‐old children born preterm.

### Autonomic nervous system

The ANS has two arms which play a significant role in maintaining homeostasis throughout the body, and exercise testing with heart rate tracking provides a unique glimpse into cardiac autonomic function (Pierpont and Voth 2004). In the heart, the sympathetic nervous system is responsible for increasing HR and increasing contractility in the ventricles. The parasympathetic arm is responsible for decreasing HR through the vagal tone and the release of acetylcholine onto cardiac pacemaker cells (Purves et al. 2001). During graded exercise to maximal exhaustion, parasympathetic tone to the heart diminishes, immediately increasing heart rate, and sympathetic tone increases throughout progressive exercise, increasing HR as demand on the skeletal muscles increases until maximal exhaustion. Immediately following maximal volitional exhaustion when exercise is stopped, parasympathetic tone is increased and sympathetic activity decreases, both resulting in heart rate decreasing for several minutes (Savin et al. 1982). This drop in HR following maximal exercise is a well‐established measure to evaluate autonomic function. A faster HRR is correlated with a more active lifestyle (Carnethon et al. 2005) and lower cardiovascular disease risk (Carnethon et al. 2012).

A slower HRR is correlated with higher cardiovascular disease risk in adults (Cole et al. 1999; Jae et al. 2008), but little is known about the implications of slow HRR in children, with one study reporting that HRR after 1 min slows with age in children (Singh et al. 2008). It has been suggested that cardiovascular disease risk is improved through increasing ANS function, and that regular physical exercise is a mechanism by which this can be achieved (Carnethon et al. 2005). In our population, physical activity was similar using a thorough and well‐established physical activity questionnaire. However, HRR was significantly slower in preterm adolescents than controls. While this may be explained by a lower VO_2max_, there was no difference in VO_2max_ relative to body weight between groups. Our results suggest that there is autonomic dysfunction present in adolescents born preterm that affects HRV and HRR, and may also affect the contractility of the heart, limiting stroke volume. In a cardiac MRI study in young adults born preterm, stroke volume and ejection fraction were lower in subjects born preterm (Lewandowski et al. 2013). We calculated maximal oxygen pulse in our study by rearranging the Fick equation, which has been used as a surrogate of stroke volume when an equal arteriovenous oxygen difference is assumed between groups (Whipp et al. 1996), and found that oxygen pulse at maximal exercise was significantly lower in the preterm group.

### Heart rate variability (R‐R intervals)

In our study, we found that resting heart rate, both derived as a calculation from calculated HR from forehead pulse oximetry and from average R‐R intervals from a resting ECG, is no different between preterm and term‐born children. While the literature studying this population is sparse, other studies in slightly younger cohorts have reported higher resting heart rates in children born preterm, though with unreported physical activity measures (Rakow et al. 2013; Bonamy et al. 2017). The lower time domain variability as expressed by the SDRR and RMSSD in preterm individuals is indicative of blunted autonomic activity in terms of cardiac heart rate response to sensory stimuli, which results in a less reactive ANS. A lower RMSSD, which is correlated with lower parasympathetic activity (DeGiorgio et al. 2010), suggests that parasympathetic activity in particular is decreased in adolescents born preterm. Lower HRV is well documented as being associated with higher cardiovascular disease risk and worse health outcomes in adults (Liao et al. 1997; Dekker et al. 2000; Evrengul et al. 2006), with evidence of a link with disease in children also (Baum et al. 2013; Kumar et al. 2017). Our findings open the door for further study of autonomic function in this otherwise healthy population.

### Exercise tolerance

Interestingly, we found that exercise tolerance is lower in preterm adolescents, despite similar physical activity scores and maximal heart rates achieved during exercise testing. We report VO_2max_ absolutely and relative to body weight for this population due to the difficulty of interpreting aerobic fitness in children of prepubertal and pubertal age, where the effect of maturation on aerobic capacity is not well understood (Armstrong et al. 2011). While there are few studies in the literature investigating fitness in children born preterm, our results agree with another study's findings showing lower exercise capacity in children born preterm (Rogers et al. 2005). Importantly, the preterm group in this prior study had significantly lower physical activity ratings than term‐born controls, while our subjects had similar physical activity scores.

### Potential mechanisms

Though the mechanisms of altered autonomic function were not elucidated in our study, there is evidence in the literature that may suggest some possibilities. Cardiac activity undergoes continuous flux in response to sensory information in order to maintain high oxygen saturation and oxygen delivery to critical tissues constant. Autonomic control of cardiac function is dictated in large part by baroreceptor feedback in the major arteries, and chemoreceptor feedback about oxygen and carbon dioxide levels in the blood is located mainly in the carotid bodies (Purves et al. 2001). During exercise, feed‐forward regulation of the ANS via supra‐medullary inputs (including baroreflex resetting) and feedback from exercising muscle plays an important role in regulation of cardiac function (Rowell 1993). Infants born extremely premature are often supported with supplemental oxygen for many weeks to months, exposing them to a relatively hyperoxic environment. In animal models of prematurity, animals are exposed to hyperoxia for several days following birth. In a rat model of premature birth, it has been shown that carotid body density is significantly lower in rats exposed to postnatal hyperoxia, coupled with reduced sensitivity of the afferent limb of the arterial chemoreceptor reflex (Bisgard et al. 2003). Preterm birth disrupts nervous system and baroreflex development in utero, and impairs the normal baroreflex growth in infants born preterm. This disruption in development appears to cause impaired autonomic responses later in infancy and into adulthood. Infants and young adults born very premature have blunted ventilatory responses to hypoxic air (Calder et al. 1994; Bates et al. 2014), and preterm infants at 5–6 months of age have lower baroreflex sensitivity than age‐matched term infants, as shown during quiet and active sleep (Witcombe et al. 2012).

### Limitations

One of the main limitations of the study is the inability to assess the mechanisms behind altered autonomic function in this population. We elected to use heart rate variability at rest and heart rate recovery from maximal exercise rather than more invasive methods due to the age of the children in the study. A second limitation in our study was the inability to assess muscle sympathetic nervous activity, a measure that would allow us to more confidently differentiate between the sympathetic and parasympathetic activity in these children. A third limitation in our study was the small sample size in each group. We may have failed to include or account for other potential confounding variables that could explain the difference in ANS function between the groups. Our future work in this population will take a more mechanistic approach, with more direct autonomic function measurements, including muscle sympathetic nervous activity measurements.

## Conclusion

In conclusion, we demonstrate autonomic dysfunction in otherwise healthy adolescent children with a history of very premature birth. These findings are particularly noteworthy for two reasons. First, these individuals are otherwise healthy, without known current cardiovascular disease. Given the association of autonomic dysfunction with increased cardiovascular risk (Thayer et al. 2010), adolescents born preterm may be at elevated risk of later cardiovascular disease. Second, previous research has suggested that infants born preterm have disrupted autonomic development that results in abnormal HRV in infancy that is no longer present by 2–3 and 6–7 years of age (Landrot et al. 2007). Our results suggest that children born preterm ages 12–14 exhibit abnormal autonomic function, consistent with a re‐emergence of disease. Further study is warranted in this population to understand both the mechanisms of dysfunction as well as the long‐term cardiovascular impacts.

## Conflict of Interest

None declared.
